# Structure-Based Discovery and Characterization of a Preclinical Drug Candidate for the Treatment of HIV-1 Infection

**DOI:** 10.3390/v14112390

**Published:** 2022-10-28

**Authors:** Dongwei Kang, Jinxuan Yang, Lingjin Kong, Ronghua Luo, Xusheng Huang, Tao Zhang, Mengdi Ma, Da Feng, Zhao Wang, Hao Fang, Peng Zhan, Yongtang Zheng, Xinyong Liu

**Affiliations:** 1 Key Laboratory of Chemical Biology (Ministry of Education), School of Pharmaceutical Sciences, Shandong University, 44 West Culture Road, Jinan 250012, China; 2 China-Belgium Collaborative Research Center for Innovative Antiviral Drugs of Shandong Province, Jinan 250012, China; 3 Key Laboratory of Bioactive Peptides of Yunnan Province/Key Laboratory of Animal Models and Human Disease Mechanisms of the Chinese Academy of Sciences, KIZ-CUHK Joint Laboratory of Bioresources and Molecular Research in Common Diseases, Center for Biosafety Mega-Science, Kunming Institute of Zoology, Chinese Academy of Sciences, Kunming 650223, China; 4 College of Traditional Chinese Medicine, Yunnan University of Chinese Medicine, Kunming 650500, China; 5 Shandong Provincial Key Laboratory of Neuroprotective Drugs, Shandong Qidu Pharmaceutical Co., Ltd., Zibo 255400, China; 6 University of Chinese Academy of Sciences, Beijing 100049, China

**Keywords:** K-5a2, HIV-1, NNRTI, pharmacodynamics, pharmacokinetics, acute toxicity

## Abstract

HIV-1 non-nucleoside reverse transcriptase inhibitors (NNRTIs) area key component of the current HIV-1 combination drug regimens. Although they exhibit potent anti-HIV-1 activity and modest toxicity, the emergence of mutant strains limits their application in clinical. Our previous research efforts contributed to the identification of compound K-5a2, which exhibits nanomolar activity in HIV-1-infected MT-4 cells. In this study, K-5a2 was shown to have a high level of anti-HIV-1 activity against various lab-adapted strains and clinical isolate strains, being comparable to ETR. Moreover, we showed the feasibility of K-5a2 as a preclinical anti-HIV-1 candidate by establishing its synergistic or additive anti-HIV-1 activity in combination with other representative anti-HIV-1 drugs and candidates. In addition, K-5a2 exhibited no inhibitory activity to the primary CYP isoforms and favorable pharmacokinetics. Taken together, its robust anti-HIV-1 potency, synergistic or additive effects with other anti-HIV drugs, and favorable pharmacokinetic and safety profiles make K-5a2 a potent alternative drug for HIV/AIDS treatment.

## 1. Introduction

**Figure 1:**
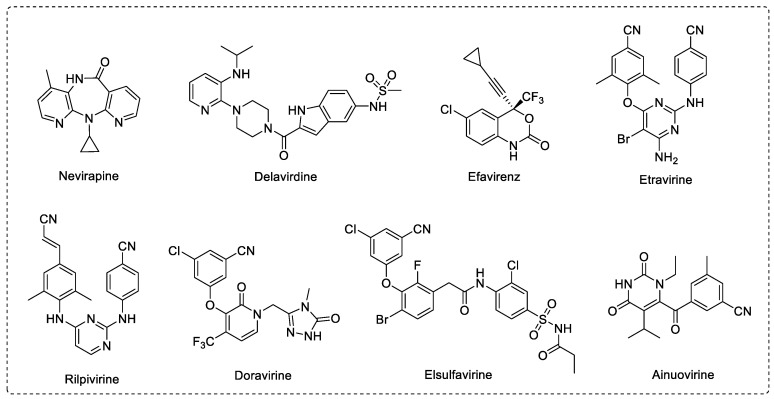
Chemical structures of approved HIV-1 NNRTI drugs.

According to the Joint United Nations Program on HIV/AIDS (UNAIDS) report in 2021, more than 38.4 million people are currently infected with human immunodeficiency virus (HIV-1), including 1.5 million new infections in 2021. Although 75% of people living with HIV received highly effective antiretroviral therapy (HAART) in 2021, 650,000 people nevertheless died of HIV-related causes [1,2]. Reverse transcriptase (RT) plays an important role in the HIV-1 replication cycle, reversing viral single-stranded RNA into double-stranded DNA [3]. HIV-1 RT is a heterodimer comprised of p66 and p51 subunits. Non-nucleoside reverse transcriptase inhibitors (NNRTIs) inhibit HIV-1 RT by binding to an allosteric hydrophobic pocket located about 10 Å from the DNA catalytic site of RT [4]. NNRTIs mainly interact with amino acid residues such as L100, K101, K103, K104, V106, V108, V179, Y181, Y188, G190, F227, W229, L234, P236, and Y318 of the NNRTI’s binding pocket (NNIBP) [5]. NNRTIs are essential components in HARRT due to the effective antiviral activity, high selectivity, and modest toxicity [6]. To date, there are more than 50 chemical types of NNRTIs, including dihydro-alkoxyl-benzyl-oxopyrimidine, diaryl ether, diarylpyrimidine (DAPY), among others [7,8]. Among them, six NNRTIs have been approved for AIDS therapy by the US Food and Drug Administration (FDA), including the first-generation drugs nevirapine (NVP), delavirdine (DLV), and efavirenz (EFV), and the second-generation drugs etravirine (ETR), rilpivirine (RPV), and doravirine (DOR) (Figure 1) [9]. Moreover, ainuovirine (ANV) and elsulfavirine (ESV) have received approval for treating HIV-1 infection in China and Russia, respectively [10,11]. However, a variety of drug-resistant mutants (such as L100I, K101P/E, K103N/S, Y181C/I/V, Y188L/I/C/V, V106A/M, F227L, E138K/R, and G190A/S) have emerged clinically, due to the high genetic diversity of HIV-1, and these mutations in RT can develop drug resistance to existing RT inhibitors [12]. Additionally, their potency is hampered by their poor pharmacokinetic profiles, which result in an increased opportunity for mutations to develop. Therefore, the continuous effort to develop novel HIV-1 NNRTIs is paramount for developing new therapeutic drugs with improved resistance profiles, low toxicity and pharmacokinetic profiles.

**Figure 2:**
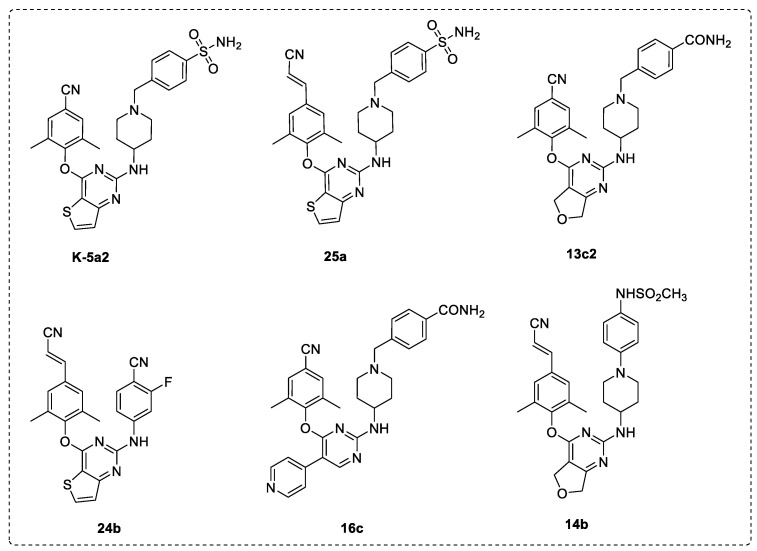
Chemical structures of HIV-1 NNRTIs K-5a2, 25a, 13c2, 24b, 16c, and 14b.

Over the past 10 years, our team has focused on the development of novel anti-HIV-1 drug candidates, based on the computer-aided drug design, synthetic organic chemistry, activity evaluation and mechanism validation, structural biology, and druggability evaluation. Previous research efforts have contributed to the discovery of a number of potent DAPY NNRTIs, such as the thiophene[3,2-d]pyrimidine derivatives K-5a2, 25a, and 24b; the dihydrofuro[3,4-d]pyrimidine derivatives 13c2 and 14b; and the 2,4,5-trisubstituted pyrimidine derivative 16c (Figure 2), which exhibit significantly improved drug resistance profiles compared to those of the approved second-generation NNRTIs ETR and RPV. In particular, compound K-5a2 was demonstrated to have optimal pharmacological properties and safety profiles, and was selected as an anti-HIV-1 drug candidate for further preclinical evaluation [13]. In this study, we describe the pharmacodynamic and pharmacokinetic milestones achieved in the preclinical research on K-5a2.

## 2. Materials and Methods

### 2.1. Ethics Statement

Ethics approval for this study and consent processes were provided by the Ethics Committee of the Kunming Institute of Zoology, Chinese Academy of Sciences (Approval Number: KIZRKX-2021-013 and SWYX-2006011).

### 2.2. Compounds and Reagents

K-5a2 and ETR were synthesized in our lab. MVC and DC521022 were provided by Hong Liu, Shanghai Institute of Materia Medica, Chinese Academy of Sciences. AZT, 3TC, and DRV were purchased from Meilunbio^®^(Dalian, China). FTC was purchased from Zhongshuo Pharmaceutical Technology Development Inc (Beijing, China). RAL was purchased from Merck Sharp & Dohme (Kenilworth, NJ, USA). TDF was purchased from Huangshi Fuertai Pharmaceutical Technology Inc (Huangshi, China). DTG was purchased from Macklin. T-20 was provided by Chengdu Shengnuo Biopharmaceutical Co., Ltd. FTC was dissolved in 0.9% sodium chloride injection and stored at −20 °C. AZT and T-20 were dissolved in RPMI-1640 media and stored at −20 °C. Other compounds were dissolved in dimethyl sulfoxide (DMSO) and stored at 4 °C.

Thiazolyl blue tetrazolium bromide, sodium chloride, Triton X-100, penicillin, potassium chloride, disodium hydrogen phosphate, Tween 20, potassium dihydrogen phosphate, PHA-P, and anti-mouse IgG (Fc specific) antibodies produced in goats were purchased from Sigma (St. Louis, MO, USA). Horseradish peroxidase-conjugated affinity pure goat anti-rabbit IgG (H+L) was purchased from KPL. Sodium dodecyl sulfate was purchase from BioFR0XX. Dimethyl sulfoxide was purchased from VWR Life Science AMRESCO. Streptomycin sulfate was purchase from Solarbio. N, N-Dimethylformamine was purchased from Chengdu Kelong Chemical Inc. IL-2 was purchased from Dalian meilunbio^®^. Lymphocyte Separation Medium (Human) was purchased from Tianjin Haoyang Biotechnology Inc. RPMI-1640 medium, DMEM medium, new born calf serum (NBCS) and fetal bovine serum (FBS) were purchased from Gibco. Skimmed milk powder produced by Inner Mongolia Yili Industrial Group Inc. was used. Anti-p24 McAb P6F4 was prepared in our laboratory. A reverse transcriptase assay kit was purchased from Sigma-Aldrich (St. Louis, MO, USA).

### 2.3. Cells and Viruses

C8166, MT-4 and TZM-bl cells were provided by the AIDS Reagent Project (MD, USA). C8166 lines were cultured in RPMI-1640 medium supplemented with 10% (*v/v*) FBS, 100 IU/mL penicillin G, and 100 μg/mL streptomycin in a humidified incubator with 5% CO_2_ at 37 °C. MT-4 cells were maintained in RPMI-1640 supplemented with 10% (*v/v*) NBCS, penicillin G (100 IU/mL), and streptomycin (100 μg/mL). TZM-bl cells were maintained in DMEM supplemented with 10% (*v/v*) FBS, penicillin G (100 IU/mL), and streptomycin (100 μg/mL). PBMCs were isolated from healthy donors by Ficoll-Hypaque density gradient centrifugation. PBMCs were maintained in RPMI-1640 supplemented with 10% (*v/v*) FBS, penicillin G (100 IU/mL), streptomycin (100 μg/mL), PHA (5 μg/mL), and IL-2 (50 U/mL) for 72 h before experiments.

Laboratory-adapted strain HIV-1_IIIB_, nucleoside reverse transcriptase inhibitor (NRTI)-resistant strain HIV-1_4755-5_, non-nucleoside reverse transcriptase inhibitor (NNRTI)-resistant strain HIV-1_A17_, fusion inhibitor (FI)-resistant strain pNL4-3_GP41(36G)V38A,N42T_, protease inhibitor (PI)-resistant strain HIV-1_RF/V82F/184V_, and integrase strand transfer inhibitor (INSTI)-resistant strain HIV-1_Yu-2(G140S/Q148H)_ were obtained from the NIH AIDS Research and Reference Reagent Program. Clinical HIV-1 isolates from local AIDS patients were propagated by co-culture with healthy PBMCs, including HIV-1_TC-1_, HIV-1_KIZ001_, and HIV-1_WAN_. All virus stocks were equally distributed and stored at −80 °C.

### 2.4. Cytotoxicity Assays

The cytotoxicity of K-5a2 on different cells (C8166, MT-4, H9/HIV-1_IIIB_, PBMCs) was determined using the MTT assay [14]. In brief, C8166, MT-4, H9/HIV-1_IIIB_ (4 × 10^5^/mL) or PBMCs (5 × 10^6^/mL) were seeded in 96-well plates containing gradient-diluted compounds, and negative control wells and blank control wells were set at 37 °C and 5% CO_2_ for three days (seven days for PBMCs). Approximately 20 µL MTT was further added to each well and incubated for 4 h. The supernatant was discarded, and 100 µL 12% SDS and 50% DMF were added. Then, the plates were incubated overnight. The absorbance value of the samples was read using a Bio Tek 800TS (Winooski, VT, USA) at 570/630 nm, and the 50% cytotoxicity concentration (CC_50_) was calculated.

### 2.5. Anti-HIV-1 Activity Assay

The antiviral assay of compounds was performed based on the viral cytopathic effect (CPE) [15]. Briefly, HIV-1_IIIB_ C8166 and compounds were co-cultured for three days. At three days post-infection, the number of syncytia in each well of the 96-well plates was counted under an inverted microscope (100×) to determine CPE. The 50% effective concentration (EC_50_) was calculated using Origin 2019b 32Bit (Northampton, MA, USA, 2019).

The anti-HIV-1_YU-2_ and HIV-1_Yu-2 (G140S/Q148H)_ activities were detected by a luciferase assay in TZM-bl cells. Briefly, cells (3 × 10^4^/well) and viruses (MOI = 0.1) were incubated in 96-well plates in the presence or absence of serial dilutions of the compound. After three days, the luciferase activity was determined using Promega’s luciferase activity assay system [15]. EC_50_ was calculated using Origin 2019b 32Bit (Northampton, MA, USA, 2019).

The activities of the anti-HIV-1 laboratory-adapted strains, resistant strains, and clinical isolate strains were measured, as mentioned earlier [15]. C8166 cells were infected with HIV-1_IIIB_, HIV-1_4755-5_, HIV-1_A17_, pNL4-3_GP41(36G)V38A,N42T_, and HIV-1_RF/V82F/184V_ with a TCID_50_ of 1000–2000. PHA-stimulated PBMCs were infected with HIV-1_TC-1_, HIV-1_KIZ001_, and HIV-1_WAN_ (MOI = 0.05). After 4 h, the cells were washed two times to remove free viruses and re-suspended in RPMI-1640 with 10% FBS. The 4 × 10^4^/well C8166 cells (5 × 10^5^/well PBMCs) were seeded in 96-well plates with different concentrations of compounds at 37 °C and 5% CO_2_. After incubation for 3-7 days, the percentage inhibition of p24 was measured by ELISA. EC_50_ was calculated using Origin 2019b 32Bit (Northampton, MA, USA, 2019).

The anti-HIV-1 activity of the compounds was tested using an MTT assay, as mentioned early [16]. In brief, MT-4 cells (4 × 10^4^/well) were infected with HIV-1_IIIB_ at different serial concentrations with a TCID_50_ of 1300. At three days post-treatment, 100 µL of drug medium was added to 96-well plates at 37 °C and 5% CO_2_ for four days. Approximately 30 µL MTT was further added to each well and incubated for 4 h. The cultural supernatant was removed, and 150 µL 12% SDS and 50% DMF were added. Then, the plates were incubated overnight. The absorbance value of the samples was read using a Bio Tek 800TS at 570/630 nm. EC_50_ was calculated using Origin 2019b 32Bit (Northampton, MA, USA, 2019).

The anti-HIV-1 laboratory-adapted strain activity was measured in the presence of human serum, as mentioned early [17]. Briefly, C8166 cells were infected with HIV-1_IIIB_ with a TCID_50_ of 2000. After 4 h, cells were washed two times to discard free viruses and resuspended in RPMI-1640 with 10% FBS. C8166 cells (50 µL, 8 × 10^5^/mL) were added in 96-well plates with different concentrations of compounds in the presence of 20% or 40% human serum at 37 °C and 5% CO_2_. After incubation for three days, the percentage inhibition of p24 was measured by ELISA. EC_50_ was calculated using Origin 2019b 32Bit (Northampton, MA, USA, 2019).

### 2.6. Reverse Transcriptase Inhibition Assays

The inhibitory effect of compounds on HIV-1 RT was detected according to the reverse transcriptase assay kit (Sigma-Aldrich) instructions. Briefly, 20 µL of recombinant HIV-1 RT (final concentration in reaction was 0.05 ng/µL) was added, 20 µL of RT inhibitor diluted in lysis buffer and reaction mixture was added per reaction tube, and the mixture was incubated for 1 h at 37 °C. The samples (60 µL) were transferred into MP module wells. The MP modules was covered with foil and incubated for 1 h at 37 °C. After rinsing with washing buffer 5 times, 200 µL of anti-DIG-POD working dilution (200 mU/mL) was added per well. The MP modules was covered with a cover foil and incubated for 1 h at 37 °C. After rinsing with washing buffer 5 times, 200 µL of ABTS substrate solution was added per well and incubated at room temperature until the color development was sufficient. The absorbance value of the samples was read using a Bio Tek 800TS reader at 405/490 nm; EC_50_ was calculated using Origin 2019b 32Bit (Northampton, MA, USA, 2019).

### 2.7. Combination Antiviral Activity Assay

The anti-HIV-1 effects of K-5a2 in combination with NNRTI-ETR, NRTIs-FTC, AZT, 3TC and TDF, INSTIs-DTG and RAL, PI-DRV, CCR5-receptor inhibitors MVC and DC521022, and FI-T-20 were detected on C8166 cells infected with HIV-1_IIIB_ or TZM-bl cells infected with HIV-1_YU-2_, as mentioned early. The inhibition of p24 antigen levels was detected by ELISA on day 3, and the luciferase activity was determined quantitatively on day 3 using Promega’s luciferase activity assay system. The combination index (CI) and dose reduction index (DRI) were calculated according to the median effect principle using CompuSyn software https://www.combosyn.com/(accessed on 11 October 2022) [18]. Virus inhibition values were input into CompuSyn software, and the ED_50_, ED_75_, ED_90_, and ED_95_ values were output by the software for further calculation. The CI value of the drug combination was calculated by the following formula:

CI value = (1*ED_50_+2*ED_75_+3*ED_90_+4*ED_95_)/10

### 2.8. Cytochrome P450 Inhibition Assay

K-5a2 was added to human liver microsomes (0.25 mg/mL), NADPH (10 mM), and various CYP enzyme probes substrate, including phenacetin (CYP1A2), diclofenac (CYP2C9), S-mephenytoin (CYP2C19), dextromethorphan (CYP2D6), and midazolam (CYP3A4M). The mixed solution was incubated at 37 °C for 10 min. As controls, α-naphthoflavone, sulfaphenazole, (+)-N-3-benzylnirvanol, quinidine, and ketoconazole were selected.

### 2.9. Pharmacokinetics Assays

K-5a2 was dissolved in polyethylene glycol (PEG) 400/normal saline (65/35, *v/v*). Then, 120 male and 120 female BALB/c mice (180–200 g) were divided into four groups, receiving intravenous (2 mg/kg) and oral (30, 60, and 120 mg/kg) doses of K-5a2, respectively. Jugular sinus blood (100 μL of blood each time) was collected at 2 min, 10 min, 30 min, 1 h, 2 h, 3 h, 4 h, 6 h, and 8 h after intravenous injection, and 5 min, 15 min, 30 min, 1 h, 2 h, 4 h, 6 h, 8 h, and 12 h after oral ingestion. The samples were centrifuged at 12,000 rpm for 3 min, and the plasma then underwent QTRAP^®^ 5500 LC/MS/MS (AB Sciex) analysis to determine the concentration of K-5a2.

### 2.10. Acute Toxicity Assays

Twenty male and 20 female BALB/c mice (18–22 g) were divided into two groups, respectively. Compound K-5a2 was suspended in PEG400/normal saline (125 mg/mL). The mice were given intragastric administration with a dosage of 5000 mg/kg after fasting for 12 h. The death and body weight of the mice were monitored.

## 3. Results and Discussion

### 3.1. Anti-HIV-1 Activity of K-5a2

**Figure 3:**
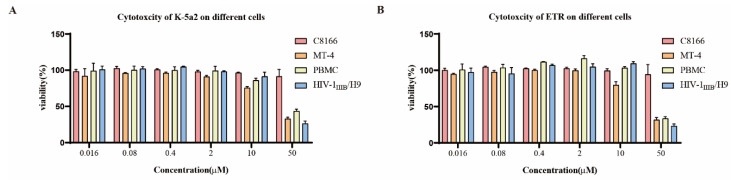
The cytotoxicity of K-5a2 (**A**) and ETR (**B**). The cytotoxicity of K-5a2 and ETR on C8166, MT-4, H9/HIV-1_IIIB_, and PBMCs was measured by MTT assay. All data represent the mean ± standard deviation for three independent replicate experiments.

**Figure 4:**
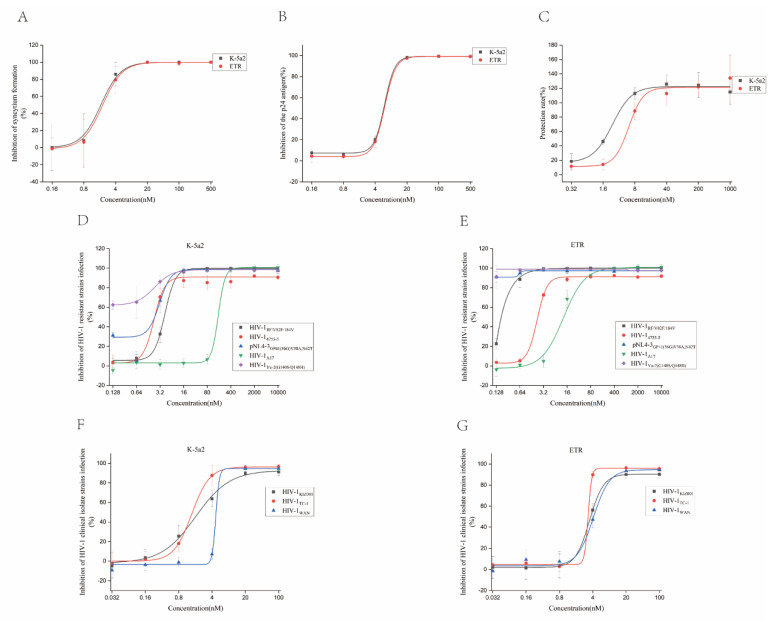
Antiviral properties of K-5a2. C8166 cells infected with HIV-1_IIIB_ and compounds were co-cultured for three days. The cytopathic effect was tested by counting the number of syncytia under an inverted microscope (**A**). Supernatants were collected at three days post-infection and p24 in the culture supernatant was analyzed by ELISA assay (**B**). The protective effects of the compounds on MT-4 cells were detected by MTT assay seven days after infection (**C**). The inhibitory effects of K-5a2 (**D**) and ETR (**E**) on HIV-1-resistant strains. C8166 cells infected by resistant strains were treated with gradient-diluted drugs for four days and p24 in the cell culture supernatants was detected by ELISA assay. The inhibitory effects of K-5a2 (**F**) and ETR (**G**) on HIV-1 clinical isolate strains. PBMCs infected by HIV-1 clinical isolates were treated with gradient-diluted drugs for seven days and cell culture supernatants were collected for p24 ELISA assay.

HIV-1 can infect human T lymphocytes, monocyte-derived macrophages, dendritic cells, and other cells in vivo and in vitro [19,20]. The drugs show different anti-HIV-1 activities in different cell types. Therefore, cell lines from multiple sources are needed to study the effects of these drugs on different HIV-1 strains. To evaluate the antiviral activity of K-5a2, the laboratory-adaptive strain HIV-1_IIIB_ was used to infect C8166 cells, and ETR was used as a control. The results are represented by EC_50_ (a mean 50% effective concentration), CC_50_ (a mean 50% cytotoxic concentration), and SI (selectivity index, CC_50_/EC_50_ ratio). As shown in Table 1, the results showed that K-5a2 had a potential inhibitory effect on HIV-1_IIIB_-induced C8166 cytopathy, with a promising activity (EC_50_ = 1.88 ± 0.87 nM), low cytotoxicity (CC_50_ > 50.00 µM) and higher selectivity index (SI > 26595.74), being comparable to those of ETR (EC_50_ = 1.74 ± 0.67 nM, CC_50_ > 50.00 µM, SI > 28735.63) (Table 1, Figure 3 and Figure 4). In C8166 cells acutely infected with HIV-1_IIIB_, K-5a2 exhibited an EC_50_ value of 6.11 ± 0.57 nM, also comparable to that of ETR (EC_50_ = 5.71 ± 1.31 nM) (Table 1, Figure 4). Meanwhile, both compounds showed no cytotoxicity at a concentration of 50.00 µM, which led to their higher SI values. These properties suggest that K-5a2 has the potential to be an anti-HIV-1 drug comparable to ETR.

MT-4 cells are also HIV-1 sensitive T cells. Unlike C8166 cells, MT-4 cells will not form syncytia after being infected with HIV-1 but will cause cell death within 6–7 days. When we evaluated K-5a2 for its inhibitory effect on HIV-1_IIIB_ replication in MT-4 cells, we found that K-5a2 has a good protective effect against HIV-1_IIIB_ infection-induced MT-4 cell death, with an EC_50_ of 1.74 ± 0.11 nM, being about three-fold more potent than that of ETR (EC_50_ = 6.15 ± 1.76 nM) (Table 1, Figure 4). The double-stranded DNA of HIV-1 in chronically infected H9 cells has been integrated into the genome of the host cell. Therefore, anti-HIV-1 drugs acting on the DNA replication, assembling, maturation, and release steps after the integration of the HIV-1 replication cycle undertake antiviral activity in chronically infected cells. However, the compounds acting on the steps of adsorption, fusion, reverse transcription, and nuclear import before the integration of the virus replication cycle did not affect the viral replication of H9 cells chronically infected with HIV-1I_IIB_. Next, we evaluated the inhibitory effect of K-5a2 on HIV-1 replication in HIV-1_IIIB_ chronically infected H9 cells. The results showed that K-5a2 and ETR could not inhibit the replication at a concentration of 50.00 µM (Table 1), suggesting that K-5a2 plays a role in suppressing the virus in the pre-integration stage.

The development of resistant mutants was rapidly observed in HIV-1-infected individuals after HHART treatment. Therefore, after studying the anti-HIV-1 activity in the laboratory-adapted strain, it was necessary to evaluate the antiviral activity of K-5a2 on the HIV-1-resistant strains, including nucleoside reverse transcriptase inhibitor (NRTI)-resistant strain HIV-1_4755-5_, NNRTI-resistant strain HIV-1_A17_, fusion inhibitor (FI)-resistant strain pNL4-3_GP41(36G)V38A,N42T_, protease inhibitor (PI)-resistant strain HIV-1_RF/V82F/184V_, and integrase strand transfer inhibitor (INSTI)-resistant strain HIV-1_Yu-2(G140S/Q148H)_. The results clearly showed that K-5a2 is a broad-spectrum inhibitor. Except HIV-1_A17_, it effectively inhibited the particle production of four other HIV-1-resistant strains (HIV-1_4755-5_, pNL4-3_GP41(36G)V38A,N42T_, HIV-1_RF/V82F/184V_, HIV-1_Yu-2(G140S/Q148H)_) in a dose-dependent manner, with EC_50_ values of 6.92 ± 7.47 nM, 2.55 ± 1.44 nM, 3.87 ± 0.98 nM, and 0.26 ± 0.28 nM, respectively (Table 1, Figure 4). However, the antiviral activity of K-5a2 against HIV-1-resistant strains was slightly lower than the positive control drug ETR.

HIV-1 clinical isolates refer to virus strains isolated from the body fluids and tissues of HIV-1-infected patients. The genetic variability and biological characteristics of clinical isolates isolated at the early stage of HIV-1 infection may reflect the virus quasispecies in patients to a certain extent [21]. According to the research on HIV molecular epidemiology, the main circulating recombinant forms (CRFs) of HIV-1 are CRF07_BC, CRF01_AE, and CRF08_BC in China [22]. Therefore, we further evaluated the potency of K-5a2 against HIV-1 clinical isolate strains that are widely prevalent in China, including HIV-1_KIZ001_ (CRF07_BC), HIV-1_TC-1_ (CRF01_AE), and HIV-1_WAN_ (CRF01_AE). Notably, K-5a2 had a broad inhibitory effect on these clinical isolates in human peripheral blood mononuclear cells (PMBCs), with EC_50_ values of 2.16 ± 0.24 nM, 2.25 ± 0.87 nM, and 5.56 ± 2.16 nM, respectively, which were equivalent to that of ETR (EC_50_ = 3.58 ± 0.62 nM, 2.27 ± 0.95 nM, and 4.58 ± 0.89 nM, respectively) (Table 1, Figure 4). Meanwhile, K-5a2 showed lower cytotoxicity in human PBMCs, with a CC_50_ value of 39.74 ± 6.29 µM (Table 1, Figure 3). These results suggest that K-5a2 has potential antiviral activity against HIV-1 clinical isolate strains comparable to that of ETR.

**Figure 5:**
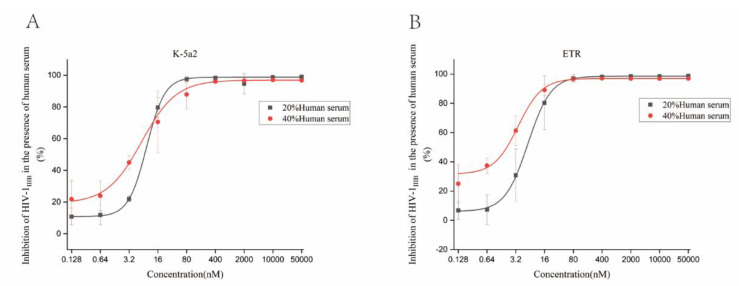
Antiviral properties of K-5a2 (**A**) and ETR (**B**) in the presence of human serum. C8166 cells infected by HIV-1_IIIB_ were treated with various concentrations of compounds in the presence of 20% or 40% human serum. After incubation for three days, the percentage inhibition of p24 was measured by ELISA assay.

A previous study demonstrated that plasma proteins, especially α1 acid glycoprotein, markedly affected the in vitro antiviral activity of the drug [23]. Therefore, the anti-HIV activity of K-5a2 in the presence of human serum was further tested. In the presence of 20% and 40% human serum, K-5a2 could inhibit HIV-1 replication in C8166 cells acutely infected with HIV-1_IIIB_, with EC_50_ values of 7.83 ± 1.73 and 7.87 ± 6.01 nM, respectively, which is comparable to the performance of ETR (EC_50_ value of 6.56 ± 4.89 and 2.09 ± 0.65 nM) (Figure 5). The results demonstrated that 20% and 40% human serum did not shield the anti-HIV-1 activity of K-5a2.

### 3.2. Inhibitory Activity to HIV-1 RT

To validate the binding target of K-5a2, it was tested for its ability to inhibit recombinant HIV-1 RT enzymes, and ETR was selected as a control drug. The results showed that K-5a2 exhibited potent inhibitory activities toward RT, with an EC_50_ of 1.11 ± 0.32 µM, which is comparable to that of ETR (EC_50_ = 1.31 ± 0.32 µM) (Table 2). These results demonstrate that the target of K-5a2 is HIV-1 RT and it acts as a classical NNRTI.

### 3.3. Combination Antiviral Activity Assay

HAART, the standard HIV-treatment regimen in clinical, usually comprises a combination of three or more anti-HIV drugs, so any late-stage preclinical candidate should be evaluated for its combined antiviral activity with other approved drugs with different targets or mechanisms. Ideally, there should be a synergistic effect, or at least it should have an addition effect. In this experiment, K-5a2 is used in combination with currently recognized and marketed anti-HIV drugs. The anti-HIV-1 activity of K-5a2 was evaluated in two drug combination studies with 11 drugs representing six categories of HAART drugs, namely, NNRTI-ETR, NRTIs-emtricitabine (FTC), zidovudine (AZT), lamivudine (3TC), tenofovir (TDF), INSTIs-dolutegravir (DTG), raltegravir (RAL), PI-darunavir (DRV), CCR5-receptor inhibitor-maraviroc (MVC), thioraviroc (DC521022), and FI-enfuvirtide (T-20). The experimental program and data processing were designed according to Zhou’s median effect principle [24]. A drug combination was defined based on the combination index (CI) as follows: CI value < 0.9, synergistic effect; CI value 0.9 to 1.1, additive effect; CI value > 1.1, antagonism effect [25]. The results demonstrated that 10 of the other drugs tested showed synergistic or additive effects with K-5a2 on HIV-1_IIIB_-infected C8166 cells, with the exception being NNRTI-ETR (Table 3). To determine the dose reduction effect (DRI) of the drug combination, the DRI was calculated. The DRI value represents the degree of dose reduction produced by the drug combination compared with the dose of each drug. The results showed the DRI value of K-5a2 combined with other drugs was 1.20–25.50-fold in the two-drug combination.

Among the combinations tested, the combination of K-5a2 and AZT had the highest DRI value, producing a 25.21-fold reduction compared to the use of K-5a2 alone (Table 3). As mentioned above, a novel anti-HIV-1 drug may be used in combination with existing HIV-1 treatments. The first-line regimens of HAART in China usually include two NRTIs and one NNRTI. The experimental results in this study indicated that K-5a2 and NRTIs have a good synergistic effect and can be considered as an alternative drug combination for antiretroviral therapy. The combination of K-5a2 and other target drugs shows a synergistic or additive effect, indicating that K-5a2 has good combination prospects and strong inhibitory activity at low doses. Our research provides reference values for clinical trials.

### 3.4. In Vitro Effects of K-5a2 on CYP Enzymatic Inhibitory Activity

Cytochrome P450 (CYP450) is the most important enzyme in the microsome mixed-function oxidase system, and more than 90% of drugs are metabolized by CYP450 in clinical [26]. Metabolic drug interactions induced and inhibited by CYP450 enzymes may alter the pharmacokinetics, efficacy, and toxicity of combined drugs [27]. ETR showed strong inhibitory activity against CYP2C9 and CYP2C19, with IC_50_ values of 0.28 μM and 0.50 μM, respectively. As depicted in Table 4, K-5a2 exhibited an IC_50_ value of 18.92 μM for CYP3A4M. In the case of CYP1A2, CYP2C9, CYP2C19, and CYP2D6, K-5a2 showed no inhibitory activity at a concentration of 50.00 μM. The results demonstrated that K-5a2 has no or weak inhibitory activity against the primary CYP isoforms, and has been associated with a reduced possibility of drug-drug interactions.

### 3.5. Pharmacokinetics of K-5a2 in Wistar Rats

**Figure 6:**
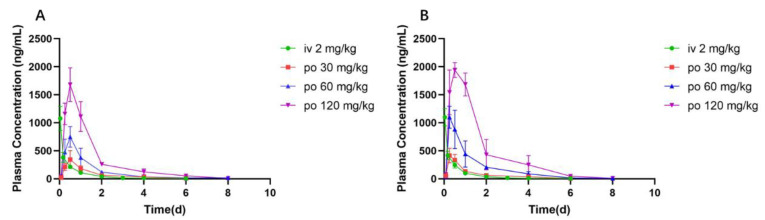
Plasma concentration–time profiles of K-5a2 in male (**A**) and female (**B**) BALB/c mice.

A detailed pharmacokinetic study of K-5a2 was carried out to determine its druggability in BALB/c mice at three oral dosage levels (30, 60 and 120 mg/kg) and after one intravenous dose (2 mg/kg) (Figure 6 and Table 5). After injection of 2 mg/kg K-5a2, the maximum concentration (Cmax) in male and female mice was 1078 μg/L and 1098 μg/L, and the half-time (T1/2) was 2.18 h and 2.35 h, respectively. After a single oral administration of K-5a2 at 30, 60, or 120 mg/kg, K-5a2 was rapidly absorbed and peaked within 0.25–0.5 h; the Cmax was linear with the dose. Moreover, oral bioavailability was 6.8 ± 1.3% and 8.5 ± 2.7% in male and female mice, respectively, which is sufficient for a drug candidate.

### 3.6. Assessment of Acute Toxicity

**Figure 7:**
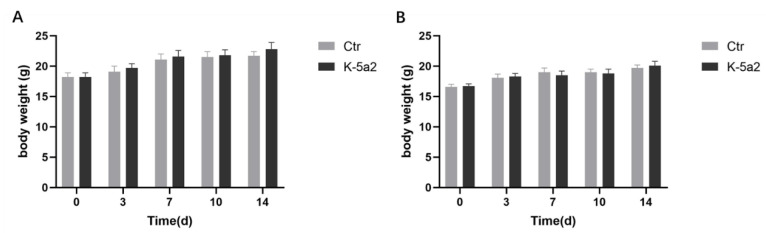
Assessment of acute toxicity. The body weight changes of male (**A**) and female (**B**) BALB/c mice after single oral doses of 5000 mg/kg.

A single-dose toxicity evaluation of K-5a2 was conducted in male and female BALB/C mice. When K-5a2 was gavaged at a dose of 5000 mg/kg, there were no obvious clinical symptoms and no obvious abnormalities in body weight (Figure 7), suggesting that the maximum tolerated dose is greater than 5000 mg/kg.

## 4. Conclusions

In general, the data presented in this study show that K-5a2, a new NNRTI, exhibits different toxicity in different cells; the results are consistent with the positive control drug ETR. K-5a2 demonstrated high anti-HIV activity against various lab-adapted strains and clinical isolate strains, which was comparable to ETR. The potent antiviral activity of K-5a2 against HIV-1 laboratory-adapted strains (HIV-1_IIIB_) and clinical isolate strains (HIV-1_TC-1_, HIV-1_KIZ00_1, and HIV-1_WAN_) in different cell lines, including C8166, MT-4, and PBMC, was also shown. In particular, the activity of K-5a2 was approximately three times more potent than that of ETR in MT-4 cells. In the combination antiviral activity assay, K-5a2 demonstrated a synergistic or additive effect when combined with other approved drugs with different mechanisms, indicating that it can be used as a main component of HAART. Moreover, K-5a2 exhibited no inhibitory activity against to the primary CYP isoforms, and showed favorable pharmacokinetic and safety properties. Taken together, the potent anti-HIV-1 activity, synergistic or additive effects with other anti-HIV-1 drugs, and favorable druggability make K-5a2 a potent alternative drug for the further development of HIV/AIDS treatment.

## Figures and Tables

**Table 1:** Summary of anti-HIV-1 activities of K-5a2 and ETR in cell cultures.

Virus	Cell	Test Method	CC_50_ (*µ*M) ^a^ (Mean ± SD)		EC_50_ (nM) ^b^ (Mean ± SD)		SI ^c^	
			K-5a2	ETR	K-5a2	ETR	K-5a2	ETR
HIV-1_IIIB_	C8166	Syncytia	>50.00 >50.00	>50.00	1.88 ± 0.87	1.74 ± 0.67	>26,595.74	>28,735.63
HIV-1_IIIB_	C8166	p24		>50.00	6.11 ± 0.57	5.71 ± 1.31	>8183.31	>8756.57
HIV-1_IIIB_	MT-4	MTT	26.63 ± 2.03	27.41 ± 5.46	1.74 ± 0.11	6.15 ± 1.76	15304.60	4456.91
HIV-1_IIIB_	H9/HIV-1_IIIB_	p24	28.05 ± 5.03	30.43 ± 1.59	>50,000.00	>50,000.00	<0.56	<0.61
HIV-1_4755-5_	C8166	p24	>50.00	>50.00	6.92 ± 7.47	3.72 ± 4.37	>7225.43	>13,440.86
HIV-1_A17_	C8166	p24	>50.00	>50.00	195.32 ± 78.66	25.92 ± 17.67	> 255.99	>1929.01
pNL4-3_GP41(36G)V38A,N42T_	C8166	p24	>50.00	>50.00	2.55 ± 1.44	<0.13	>19,607.84	>390,625.00
HIV-1_RF/V82F/184V_	C8166	p24	>50.00	>50.00	3.87 ± 0.98	0.21 ± 0.05	>12,919.90	>238,095.24
HIV-1_Yu-2(G140S/Q148H)_	TZM-bl	luciferase	-	-	0.26 ± 0.28	<0.13	-	-
HIV-1_TC-1_	PBMC	p24	39.74 ± 6.29	34.45 ± 3.06 34.45 ± 3.06 34.45 ± 3.06	2.25 ± 0.87	2.27 ± 0.95	17,662.22	15,176.21
HIV-1_KIZ001_	PBMC	p24	39.74 ± 6.29		2.16 ± 0.24	3.58 ± 0.62	18,398.15	9622.91
HIV-1_WAN_	PBMC	p24	39.74 ± 6.29		5.56 ± 2.16	4.58 ± 0.89	7147.48	7521.83

^a^ CC_50_: the 50% cytotoxicity concentration. ^b^ EC_50_: the 50% effective concentration. ^c^ SI: CC_50_/EC_50_. All data represent the mean ± SD deviation for three independent replicate experiments.

**Table 2:** Inhibitory activity of K-5a2 against HIV-1 RT.

Compounds	EC_50_ (*µ*M) (Mean ± SD, *n* = 2) ^a^
K-5a2	1.11 ± 0.32
ETR	1.31 ± 0.32

^a^ EC_50_: the 50% effective concentration. All data represent the mean ± SD deviation for two independent replicate experiments.

**Table 3:** Effect of K-5a2 combined with antiviral drugs on antiviral activity of C8166 or TZM-bl cells in vitro.

Drug Type	Drugs	Single Drug	DRI Value ^a^				CI Value ^f^	Description
			ED_50_^b^	ED_75_^c^	ED_90_^d^	ED_95_^e^		
NNRTIs	K-5a2+ETR	K-5a2	1.56 ± 0.38	1.47 ± 0.24	1.40 ± 0.12	1.35 ± 0.07	1.19 ± 0.06	antagonism
		ETR	2.71 ± 0.49	2.44 ± 0.52	2.20 ± 0.58	2.06 ± 0.61		
NRTIs	K-5a2+FTC	K-5a2	6.99 ± 0.99	5.00 ± 0.71	3.58 ± 0.51	2.85 ± 0.41	0.66 ± 0.12	synergistic
		FTC	2.57 ± 0.05	2.38 ± 0.10	2.22 ± 0.23	2.11 ± 0.32		
	K-5a2+AZT	K-5a2	25.21 ± 26.15	13.26 ± 9.86	7.60 ± 3.58	5.48 ± 2.08	0.38 ± 0.20	synergistic
		AZT	4.71 ± 3.77	4.88 ± 2.30	5.60 ± 2.33	6.52 ± 3.94		
	K-5a2+3TC	K-5a2	2.83 ± 0.37	2.79 ± 0.14	2.81 ± 0.58	2.85 ± 0.93	0.69 ± 0.12	synergistic
		3TC	2.46 ± 0.38	2.83 ± 0.52	3.28 ± 0.80	3.64 ± 1.06		
	K-5a2+TDF	K-5a2	2.48 ± 1.05	2.30 ± 0.73	2.15 ± 0.47	2.06 ± 0.33	0.78 ± 0.12	synergistic
		TDF	3.08 ± 1.25	3.22 ± 0.71	3.46 ± 0.35	3.69 ± 0.66		
INSTIs	K-5a2+DTG	K-5a2	1.54 ± 0.69	1.69 ± 0.37	1.94 ± 0.10	2.19 ± 0.50	0.96 ± 0.03	additive
		DTG	2.28 ± 0.49	2.37 ± 0.28	2.50 ± 0.41	2.61 ± 0.68		
	K-5a2+RAL	K-5a2	2.43 ± 1.08	2.45 ± 0.95	2.47 ± 0.85	2.49 ± 0.79	0.77 ± 0.28	synergistic
		RAL	2.73 ± 0.70	3.08 ± 1.11	3.50 ± 1.64	3.83 ± 2.07		
PIs	K-5a2+DRV	K-5a2	2.04 ± 0.64	2.26 ± 0.28	2.57 ± 0.19	2.85 ± 0.57	0.72 ± 0.08	synergistic
		DRV	2.39 ± 0.31	2.82 ± 0.21	3.41 ± 0.86	3.93 ± 1.44		
CCR5-receptor inhibitors	K-5a2+MVC	K-5a2	1.32 ± 0.19	1.30 ± 0.17	1.28 ± 0.15	1.27 ± 0.15	0.99 ± 0.08	additive
		MVC	4.13 ± 0.24	4.58 ± 0.50	5.10 ± 0.83	5.49 ± 1.09		
	K-5a2+DC521022	K-5a2	1.52 ± 0.51	1.61 ± 0.50	1.71 ± 0.48	1.78 ± 0.47	0.86 ± 0.08	synergistic
		DC521022	4.09 ± 2.41	5.41 ± 4.21	7.32 ± 6.96	9.08 ± 9.61		
FIs	K-5a2+T-20	K-5a2	5.69 ± 1.18	5.13 ± 0.38	4.68 ± 0.43	4.43 ± 0.75	0.66 ± 0.02	synergistic
		T-20	1.85 ± 0.85	2.05 ± 0.42	2.37 ± 0.18	2.69 ± 0.66		

^a^ DRI: dose reduction index. ^b^ ED_50_: the 50% effective dose. ^c^ ED_75_: the 75% effective dose. ^d^ ED_90_: the 90% effective dose. ^e^ ED_95_: the 95% effective dose. ^f^ CI: combination index. All data represent the mean ± SD deviation for three independent replicate experiments.

**Table 4:** CYP inhibitory activity of K-5a2.

Compounds	IC_50_ (*μ*M)				
	CYP1A2	CYP2C9	CYP2C19	CYP2D6	CYP3A4M
K-5a2	>50.00	>50.00	>50.00	>50.00	18.92
ETR	7.48	0.28	0.50	12.02	41.34
*α*-Naphthoflavone	0.19	-	-	-	-
Sulfaphenazole	-	0.66	-	-	-
(+)-*N*-3-benzylnirvanol	-	-	0.26	-	-
Quinidine	-	-	-	0.16	-
Ketoconazole	-	-	-	-	0.04

**Table 5:** Pharmacokinetic profile of K-5a2.

Parameter	Unit	iv (2 mg/kg)		po (30 mg/kg)		po (60 mg/kg)		po (120 mg/kg)	
		Male	Female	Male	Female	Male	Female	Male	Female
AUC_(0–t)_	μg·L^−1^·h	454	461	482	488	968	1423	2465	3579
t_1/2z_	h	2.18	2.35	1.04	1.09	1.42	1.19	1.75	0.82
T_max_	h	0.033	0.033	0.50	0.25	0.50	0.25	0.50	0.50
Vd	L·kg^−1^	13.3	14.1	91.4	95.0	125.1	71.9	120.2	39.7
CL	L·h^−1^·kg^−1^	4.23	4.15	60.93	60.44	61.26	41.85	47.48	33.43
C_max_	*μ*g·L^−1^	1078.7	1098.3	344.7	416.2	749.0	1098.9	1681.5	1941.3
*F*	%	-	-	7.08	7.05	7.10	10.24	9.05	12.92

## Data Availability

The data presented in this study are available in the present article.
